# Progress and challenges for the application of machine learning for neglected tropical diseases

**DOI:** 10.12688/f1000research.129064.2

**Published:** 2025-05-20

**Authors:** ChungYuen Khew, Rahmad Akbar, Norfarhan Mohd-Assaad

**Affiliations:** 1 Department of Applied Physics, Faculty of Science and Technology, Universiti Kebangsaan Malaysia, Bangi, Selangor, 43600, Malaysia; 2 Department of Immunology, University of Oslo, Oslo, Oslo, 0372, Norway; 3 Institute of Systems Biology (INBIOSIS), Universiti Kebangsaan Malaysia, Bangi, Selangor, 43600, Malaysia

**Keywords:** Neglected Tropical Diseases, Machine Learning, Drug Development, Drug Discovery.

## Abstract

Neglected tropical diseases (NTDs) continue to affect the livelihood of individuals in countries in the Southeast Asia and Western Pacific region. These diseases have been long existing and have caused devastating health problems and economic decline to people in low- and middle-income (developing) countries. An estimated 1.7 billion of the world’s population suffer one or more NTDs annually, this puts approximately one in five individuals at risk for NTDs. In addition to health and social impact, NTDs inflict significant financial burden to patients, close relatives, and are responsible for billions of dollars lost in revenue from reduced labor productivity in developing countries alone. There is an urgent need to better improve the control and eradication or elimination efforts towards NTDs. This can be achieved by utilizing machine learning tools to better the surveillance, prediction and detection program, and combat NTDs through the discovery of new therapeutics against these pathogens. This review surveys the current application of machine learning tools for NTDs and the challenges to elevate the state-of-the-art of NTDs surveillance, management, and treatment.

**Abbreviations**
AAC: Amino Acid Composition
ACC: Scoring based on maximum accuracy
ACODAT: Autonomous Cycle Of Data Analysis Tasks
Adaboost: Adapative Boosting
AF2: AlphaFold2
AI: Artificial Intelligence
AI-DP
: AI-based Digital Pathology
ALM: Antibody Language Model
ANN: Artificial Neural Network
AntiBERTa: Antibody-specific Bidirectional Encoder Representation from Transformers
ARACON: Asian Rabies Control Network
ARIMA: Autoregressive Integrated Moving Average
ASEAN: Association of Southeast Asian Nations
AUC: area under the ROC Curve
BERT: Bidirectional Encoder Representations from Transformers
C-ImmSim
: C-language version of the IMMune system SIMulator
CASP14: Critical Assessment of Structure Prediction 14th edition
CDRs: complementarity-determining regions
CNN: Convolutional neural network
CNS: Central Nervous System
Cryo-EM
: cryogenic electron microscopy
CT: Computed Tomography
CTD: composition, transition and distribution
CTDC: CTD-composition
CTDD: CTD-distribution
CTDT: CTD-trancomposition
CV: Computer Vision
CYD-TDV
: chimeric yellow fever virus-DENV tetravalent dengue vaccine
DALYs: Disability-Adjusted Life Years
DBLP: Digital Bibliography and Library Project
DENV: Dengue Virus
DF: dengue fever
DHF: dengue haemorrhagic fever
DHS: Demographic and Health Surveys
DL: Deep Learning
DNA: deoxyribonucleic acid
DPC: dipeptide composition
DSS: dengue shock syndrome
DT: Decision Tree
EPG: eggs-per-gram
ECDC: The European Centre for Disease Prevention and Control
ESM-1: endothelial cell specific molecule-1
ESPEN: Expanded Special Project for the Elimination of NTDS
FCM: Fuzzy c-Mean
FFBPNN: Feed Forward Back Propagation Neural Network
GAAC: Grouped Amino Acid Composition
GAELF: Global Alliance to Eliminate Lymphatic Filariasis
GAHI: Global Atlas of Helminth Infections
GARC: Global Alliance for Rabies Control
GBD: Global Burden of Disease Study
GBM: Gradient Boosting Machine
GEMME: Global Epistatic Model Predicting Mutational Effects
GHDx: Global Health Data Exchange
GHE: Global Health Estimates
GHO: WHO Global Health Observatory
GLEAN: Global Leptospirosis Environment Action Network
GLM: Generalized Linear Model
GPELF: Global Programme to Eliminate Lymphatic Filariasis
GWR: Geographically Weighted Regression
iAMAP-SCM
: identification of antimalarial peptides with an interpretable scoring card method
IMGT: International IMmunoGeneTics information system
kNN: k-Nearest Neighbour
LAD Tree: Least Absolute Deviation Tree
LAMP: loop-mediated isothermal amplification
LF: Lymphatic Filariasis
LM: Language Models
LMT: Logistic Model Tree
LOOCV: Leave-one-out Cross Validation
LOPOV: Leave-one-pathogen-out Validation
LR: Logistic Regression
LSTM-ATT
: Attention-Enchanced Long Short-Term Memory
MAE: Mean Absolute Value
MAP: Malaria Atlas Project
MARS: Multiple Adaptive Regression Splines
MERACON: Middle East, Eastern Europe, Central Asia and North Africa Rabies Control Network
MCC: Matthew’s coefficient correlation
MDA: mass drug administration
ML: Machine Learning
MLP: Multilayer Perceptron
MLPNN: Multilayer Perceptron Neural Network
MMDP: Morbidity Management and Disability Prevention
MODWT: Maximal Overlapping Version of Discrete Wavelet Transformation
MRI: Magnetic Resonance Imaging
MSA: multiple sequence alignments
MSE: Mean Square Error
N5CV: Nested 5-fold cross-validation
NB: Naïve Bayes
NCC: neurocysticercosis
NLP: Natural Language Processing
NMR: Nuclear Magnetic Resonance spectroscopy
NN: Neural Network
NTDs: Neglected Tropical Diseases
OAS: Observed Antibody Space
PARACON: Pan-African Rabies Control Network
PC: preventive chemotherapy
PCR: polymerase chain reaction
PET: Positron-emmission tomography
PLM: Protein Language Model
PLOS: Public Library of Science
PLSLR: Partial Least Squares Regression
PNN: Probabilistic Neural Network
PPI: Protein-Protein Interaction
ProABC-2: Prediction of AntiBody contacts v2
QSAR: Quantitative structure-activity relationships
RF: Random Forest
RFE: Recursive Feature Elimination
RGN2: Recurrent Geometric Network
RMSE: Root Mean Square Error
ROC: Receiver operating characteristics curve
RT: Regression Tree
RV: reverse vaccinology
SARS-Cov-2: Severe Acute Respiratory Syndrome Coronavirus 2
SHAP: Shapley Additive Explanation
SEA: Southeast Asia
SIT: Sterile Insect Techniques
STHs: soil-transmitted helminthiasis
STRING: Search Tool for the Retrieval of Interacting Genes/Proteins
SVM: Support Vector Machine
SVR: Support Vector Regression
TAS: Transmission Assessment Survey
TPC: tripeptide composition
WASH: Wash, Sanitation And Hygiene
Vaxign-ML
: supervised machine learning reverse vaccinology model for improved prediction of bacterial protective antigens
VESPA: Variant Effect Score Prediction

## Introduction

### Neglected tropical diseases (NTDs)

Communicable diseases are illnesses brought on by pathogens such as bacteria, viruses, parasitic worms, and fungi that can be contracted easily from contaminated surfaces, water, air droplets, air, bites from vector organisms, and direct contact with infected individuals. A recent Coronavirus disease (COVID-19) pandemic caused by the novel coronavirus Severe Acute Respiratory Syndrome Coronavirus 2 (SARS-CoV-2), with 623,470,447 confirmed cases of COVID-19, and 6,551,678 reported deaths, as of 20 ^th^ October 2022 ( https://covid19.who.int/), underlines the need for highly efficient approaches to manage, surveil, and develop new treatments for communicable diseases. Unlike the COVID-19 pandemic, a ‘silent yet horrific endemic’ caused by a diverse group of diseases affecting more than 149 countries and with more than 1.7 billion of infected individuals worldwide does not receive the same amount of attention. The diverse group of diseases was coined by Peter Hotez and colleagues as ‘neglected tropical diseases’ (NTDs) as they do not receive the same urgency, in neither treatments nor surveillance, as other diseases such as AIDS, malaria, and tuberculosis ( Winkler et al. 2018; Parashar et al. 2024). The World Health Organization (WHO) first established a list of 17 “official” NTDs ( World Health Organization 2010). Later in 2017, an addition of 3 disease conditions were made at the end of the 10 ^th^ meeting of the Strategic and Technical Advisory Group for Neglected Tropical Diseases ( World Health Organization 2017). 20 NTDs recognized by WHO are shown in Table 1.

**Table 1.** List of NTDs recognized by WHO.

Category	Disease
i. Protozoan infections	1. Chagas disease
	2. Human African trypanosomiasis
	3. Leishmaniasis
ii. Helminth infections	4. Taeniasis/Cysticercosis
	5. Dracunculiasis
	6. Echinococcosis
	7. Foodborne trematodes
	8. Lymphatic filariasis (LF)
	9. Onchocerciasis
	10. Schistosomiasis
	11. Soil-transmitted helminthiases - Ascariasis - Hookworm diseases - Trichuriasis
iii. Bacterial infections	12. Buruli ulcer
	13. Leprosy
	14. Trachoma
	15. Yaws
iv. Viral infections	16. Dengue and chikungunya fevers
	17. Rabies
v. Fungal Infections *	18. Mycetoma, chromoblastomycosis, and other deep mycosis
vi. Ectoparasitic infections *	19. Scabies, Myiasis
vii. Venom *	20. Snakebite envenoming

Source: https://www.who.int/health-topics/neglected-tropical-diseases#tab=tab_1.

^*^ Newly added diseases conditions into the NTD list prior to the outcome of the 10 ^th^ meeting of the Strategic and Technical Advisory Group for Neglected Tropical Diseases.

### Disability-adjusted life year (DALY) impact of NTDs

High incidences of NTDs were commonly reported from tropical countries due to its optimal humidity and climate for the pathogens to thrive. African and Asian countries with low and moderate incomes tend to lack proper access to clean water and waste management, thus greatly contributing to the spread of NTDs among women and children. To measure the extent of devastation caused by NTDs, the disability-adjusted life year (DALY; one DALY represents the loss of the equivalent of one year of full health) metric was introduced as a means to quantify the overall burden of disease borne by individuals ( Mitra & Mawson 2017; Lin et al. 2022). DALYs for a disease or health condition are the sum of years of life lost (YLLs) due to premature mortality and years of healthy life lost due to disability (YLDs) due to prevalent cases of the disease or health condition in a population ( Vinkeles Melchers et al. 2021). Based on the data collected by WHO, we were able to summarize the global burden for 14 of the 20 NTDs as estimated by DALYs in Table 2 below. Global burden for five of the highest estimated DALY burden NTDs are rabies (2.635 million years), dengue (1.952 million years), soil-transmitted helminthiases (STHs) (1.943 million years), schistosomiasis (1.628 million years), and lymphatic filariasis (LF) (1.616 million years). In another report from the Global Burden of Disease Study 2019, the estimated DALYs of NTDs was 15.142 million years, with the highest burden for dengue, followed by STHs, schistosomiasis, LF, and cysticercosis ( Vos et al. 2020). When grouped based on category, helminth infections are responsible for the highest DALYs burden and widespread debilitating illnesses, with STHs as the leading cause of human helminthiases ( Vos et al. 2020; World Health Organization 2020).

**Table 2.** Global burden for 14 of 20 NTDs estimated in Disability-Adjusted Life Years (DALYs).

Diseases	WHO Global Health Estimate (2019) ^a^			Global Burden Disease Study (2019) ^b^
	YLLs	YLDs	DALYs	DALYs
Rabies	2,634,634	146	2,634,780	782,000
Dengue	1,413,126	539,243	1,952,369	2,380,000
Soil-transmitted helminthiasis:	170,570	1,772,444	1,943,014	1,970,000
a) Ascariasis	170,523	578,095	748,618	754,000
b) Trichuriasis	0	231,942	231,942	236,000
c) Hookworm disease	47	962,407	962,454	984,000
Schistosomiasis	428,141	1,199,703	1,627,844	1,640,000
Lymphatic filariasis	105	1,616,028	1,616,133	1,630,000
Onchocerciasis	12	1,209,707	1,209,720	1,230,000
Cysticercosis	348,191	639,618	987,809	1,370,000
Foodborne trematodes	0	805,406	805,406	780,000
Leishmaniasis	420,844	301,433	722,278	697,000
Echinococcosis	387,710	73,213	460,923	122,000
Chagas disease	159,632	57,482	217,113	275,000
Trachoma	22	194,369	194,391	181,000
Human African Trypanosomiasis	101,091	1,009	102,099	82,600
Leprosy	7,553	28,884	36,437	28,800
Total	6,242,201	10,211,129	16,453,330	15,142,400

^a^ World Health Organization (2020).

^b^ Vos *et al.* (2020).

### NTDs and other diseases that are of public health concern in SEA region

To provide a focused understanding of the disproportionate burden of NTDs in Southeast Asia (SEA), we summarized the DALY for each WHO region in Table 3. Based on the DALYs estimate by cause and WHO region in 2019, dengue has the highest burden of 1.510 million DALYs, followed by LF (1.029 million years), STHs (0.616 million years), rabies (0.455 million years), and cysticercosis (0.109 million years) in WHO-SEA Region. As previously highlighted, the SEA region is endemic to several vector-borne diseases such as arboviral diseases, cysticercosis, and rabies, which post significant health challenges if left undiagnosed and untreated. While melioidosis, leptospirosis, and malaria (other diseases of public health concern) not being officially recognized as NTDs by the WHO and other international agencies, it’s persistent burden and neglect, specifically in the Southeast Asia region, remain as significant contributors to the public health burden in SEA, warrants them among the major contributors to the public health concern. In this paper, we focus on diseases form the WHO’s NTD list that are of alarming concern in SEA region, as well as other diseases that surpass the impact of NTDs and present substantial public health challenges.

**Table 3.** The disability-adjusted life year (DALY) Estimates for 14 of 20 NTDS by WHO Region.

Diseases	WHO Regions					
	Africa	America	Eastern Mediterranean	Europe	Southeast Asia	Western Pacific
Rabies	1,328,185	5,548	107,891	10,279	454,966	727,911
Dengue	49,712	76,976	104,969	24	1,509,709	210,980
Soil-transmitted helminthiasis:	819,390	104,329	175,762	5,203	616,183	222,148
a) Ascariasis	243,250	35,320	111,253	930	303,462	54,403
b) Trichuriasis	57,841	23,285	4,445	100	74,341	71,931
c) Hookworm disease	518,299	45,724	60,064	4,173	238,380	95,814
Schistosomiasis	1,314,151	89,976	128,868	894	488	93,468
Lymphatic filariasis	412,847	17,432	22,406	0	1,028,642	134,805
Onchocerciasis	1,204,501	483	4,736	0	0	0
Cysticercosis	371,541	126,959	829	3,401	109,144	375,935
Foodborne trematodes	0	52,454	17,980	1,839	12,277	720,857
Leishmaniasis	271,033	36,327	341,843	3,464	69,590	21
Echinococcosis	156,691	23,966	132,135	39,846	47,016	61,269
Chagas disease	0	216,428	0	649	0	36
Trachoma	79,158	1,083	50,443	22	50,603	13,082
Human African Trypanosomiasis	102,066	34	0	0	0	0
Leprosy	6,059	8,786	1,712	61	16,988	2,831
Total	6,934,724	865,110	1,265,336	70,885	4,531,789	2,785,491

Source: World Health Organization (2020).

Dengue fever is a mosquito-borne viral disease spread to humans by the bites of infected female mosquitoes, primarily of the *Aedes aegypti* species ( Malavige et al. 2023). The disease is caused by members of the genus *Flavivirus*, within the *Flaviviridae* family ( Molyneux 2019). Members of *Flavivirus* are responsible for a wide range of other deadly infections, whereby the virus that causes dengue comprises four different but closely related serotypes: DENV-1, DENV-2, DENV-3 and DENV-4 ( Côrtes et al. 2023). These viruses are capable of causing illnesses of dengue fever (DF), dengue haemorrhagic fever (DHF), and dengue shock syndrome (DSS). In the past two decades, a 10-fold increase of 505,430 cases in 2000 to more than 5.2 million dengue cases were observed in 2019 ( World Health Organization 2021). GBD 2019 estimated 2.38 million DALYs lost and age-standardized rates of 32.1 DALYs per 100,000 (95% UI 11.1 – 44.1) ( Vos et al. 2020).

Lymphatic filariasis (LF) is caused by a group of helminths (roundworm) from the family of *Filariodidea* that reside in the lymphatic systems of humans ( Bizhani et al. 2021). *Wuchereria bancrofti* is the primary infectious agent globally, with *Brugia malayi* and *Brugia timori* as runner-ups for LF infections ( Lin et al. 2022). Mosquitoes from the genera of *Anopheles*, *Culex*, *Aedes*, and *Mansonia* are responsible for the spread of LF ( Kermelita et al. 2024). Like many mosquito-borne diseases, mosquitoes picked up microfilariae when feeding on an infected LF individual. Then, the parasite develops within the mosquito and subsequently infects the next victim on the subsequent blood meal ( Bizhani et al. 2021). The devastating harm of having adult parasite nests and microfilaria discharged into the circulation of a person's lymphatic vessels leads to disease morbidity. Functionally impaired lymphatic systems lead to the manifestation of lymphoedema (elephantiasis), hence the enlarged state of the patient's limbs. Physically impaired people experience years of disability, stigmatization, and mental health comorbidity ( Abela-Ridder et al. 2020). GBD 2019 estimated a DALYs burden of 1.630 million years, with age-standardized rates of 20.7 DALYs per 100,000 (95% UI 12.2 – 34.7) ( Vos et al. 2020).

Soil-transmitted helminths (STHs) typically infect the host’s gastrointestinal region. WHO focuses on three STHs illnesses namely ascariasis, trichuriasis, and hookworm diseases. Ascariasis and trichuriasis are spread through ingestion of fecal-contaminated food or water ( Muñoz-Antoli et al. 2022). Hookworm infection can be contracted when going barefoot on contaminated ground, in which the larvae develop into a form that enables them to pierce through human skin ( Caldrer et al. 2022). Studies revealed that the presence of high prevalence of STH are due to poor sociodemographic and socioeconomic status, especially in rural areas with poor infrastructure facilities, improper sewage and waste management, inadequate water supply, prolonged direct contact with soil such as walking barefooted, and poor sanitation and self-hygiene ( Hussein et al. 2022; Ali et al. 2020). GBD 2019 estimated similar figures to WHO GHE 2019, with DALYs burden of 1.970 million years and age-standardized rates of 26.6 DALYs per 100,000 (95% UI 17 – 40.5) ( Vos et al. 2020).

Rabies is a zoonotic disease from the *Lyssavirus* genus in the *Rhabdoviridae* family that has the capability to infect all mammalian lifeforms ( Condori et al. 2020). The disease is a lyssavirus-induced acute, progressive encephalitis and has caused a high count of human mortality and economic consequences ( World Organisation for Animal Health 2008; Alemar Ali, 2022). Once infected, the disease often results in inflammation of the brain's active tissues, causing headaches, stiff necks, light sensitivity, mental confusion, and seizures ( Chen et al. 2025). Bite wounds or contamination of open cut wounds with infected saliva are primarily how the virus is transmitted ( World Organisation for Animal Health 2008). Individuals in close contact with any suspected rabid animals should seek for post-exposure prophylaxis (PEP) treatment as a necessary means to prevent human rabies infection ( Mohammad Basir et al. 2025). The virus proliferates on the central nervous system (CNS) and thus laboratory techniques on sample processing are focused at said area ( Chen et al. 2025). GBD 2019 estimated a DALYs burden of 782,000 years with age-standardized rates of 10.6 DALYs per 100,000 (95% UI 4.4 – 14.7) ( Vos et al. 2020).

Cysticercosis is a cestode infection in both humans and porcine, caused by parasitic larval form (cysticercus) of *Taenia solium*, after consuming food or water contaminated with feces containing *T. solium* eggs (fecal-oral contamination) ( Kabululu et al. 2023). When the eggs are consumed, they hatch in the colon, releasing oncospheres that breach the intestinal wall and enter the bloodstream. From there, they migrate to various tissues and organs (including the muscles, skin, eyes, and central nervous system), where they develop into cysticerci ( Galipó et al. 2021). Cysticercosis can still develop in communities that do not consume pork or share habitats with pigs since this disease is spread by swallowing *T. solium* eggs that are shed in the feces of a human *T. solium* carrier. Development of parasitic cysts in the brain or central nervous system is referred to as neurocysticercosis (NCC) ( Mlowe et al., 2024). GBD 2019 estimated a higher DALYs burden of 1.370 million years with age-standardized rates of 16.8 DALYs per 100,000 (95% UI 10.7 – 23.9) ( Vos et al. 2020).

Melioidosis or Whitmore’s disease is a bacterial infection caused by *Burkholderia pseudomallei*, a soil- and water-borne Gram-negative bacterium capable of causing illness ranging from an acute or chronic localized infection to a widespread septicemic infection in multiple organs ( Cheng & Currie 2005; Narne et al. 2024). Cases of melioidosis are frequently reported in endemic countries such as Africa, Australia, China India, Middle East, and Southeast Asia (typically Malaysia, Singapore, and Thailand) ( Rout et al. 2025). Since its discovery in 1912, the bacteria still remains a topic of discussion among researchers due to its zoonotic nature, limited therapeutic options with no available vaccines till date ( Nyanasegran et al. 2023). Moreover, *B. pseudomallei* was designated as a Tier 1 select agent given its biothreat potential including high morbidity and mortality rates in low infectious doses, multidrug antibiotic resistance, and the amenability to be aerosolized ( Gassiep et al. 2021). Melioidosis infection can be acquired through many routes with skin inoculation and inhalation or ingestion of contaminated water and air droplets to be the leading cause. The disease mimics the signs and symptoms of other diseases (tuberculosis, malaria, dengue) often complicating the accurate diagnosis for the disease ( Bzdyl et al. 2022). As such, a study on the burden of melioidosis has estimated approximately 165,000 cases with a mortality rate greater than 50% (89,000 deaths) globally in 2015 ( Limmathurotsakul et al. 2016; Meumann et al. 2024). There is no available data reporting DALYs estimate for melioidosis from WHO GHE 2019 and GBD 2019. Up-to-date presently available global burden of melioidosis in 2015 by Birnie et al. (2019) and Meumann et al. (2024) described an estimated 4.64 million DALYs which surpassed all 16 other NTDs.

Leptospirosis is a zoonotic disease caused by a lethal bacterium of the genus *Leptospira* ( Narkkul et al. 2021). The bacteria resides in the host’s kidney, completing its lifecycle before being shed in the urine. Molecular serotyping studieshave concluded more than 250 serovars, which can be further segregated into 30 serogroups ( Hagedoorn et al. 2024). Various wild and domesticated mammals are suitable host reservoirs for *Leptospira* spp. In the city; rodents are the most important host sources of leptospirosis infection as they can persistently shed pathogenic *Leptospira* spp. to the environment throughout their lifecycle without any clinical manifestations ( Urbanskas, Karvelienė & Radzijevskaja 2022). According to Sayanthi and Susanna (2024), *Leptospira* spp. can survive for up to 20 months at 30°C and up to 10 months at 4°C. Human individuals can contract the illness through direct contact with *Leptospira*-contaminated urine, water, and wet soil ( Sun, Liu & Yan 2020). Pathogenic *Leptospira* spp. infections may be asymptomatic or exhibit a variety of clinical symptoms, from acute febrile sickness to severely defined multiple organ failures, mimicking symptoms of other threatening diseases such as dengue, influenza, and malaria ( Azevedo et al. 2023). There is no accurate data available regarding the burden of leptospirosis from WHO GHE 2019 and GBD 2019, hence a model study calculated that there are roughly 1.03 million incidents of leptospirosis annually worldwide, of which 5.72% (58,900) result in mortality ( Costa et al. 2015; Agampodi et al. 2023). Additionally, those figures were incorporated to estimate the global burden of leptospirosis in terms of DALYs which were predicted to be at 2.90 million DALYs annually, representing incidence of 41.8 DALYs per 100,000 population (UI 18.1 – 65.5) ( Torgerson et al. 2015; Wainaina et al. 2024).

Mosquito bites from infected females *Anopheles* spp. deliver the deadly parasites that cause malaria, which is a long-standing disease. The causative agent for malaria is a group of unicellular protozoan parasites originating from the *Plasmodium* genus ( Sato 2021). All *Plasmodium* spp. are capable of infecting malaria but to a specific range of host, with *P. falciparum*, *P. vivax*, *P. malariae*, *P. ovale*, and *P. knowlesi* as natural vectors for malaria among humans ( Boundenga et al. 2024). Furthermore, *P. falciparum* is the most lethal and prevalent malaria parasite in Africa, whereas *P. vivax* is the most common malaria infection outside of Sub-Saharan Africa ( Sato 2021). In 2020, it was predicted that there were 241 million cases of malaria, resulting in 627,000 deaths ( Singh et al. 2022). In the same report, malaria has posed a great threat to about half of the world's population, with sub-Saharan Africa reporting the greatest number of cases and fatalities. The burden of malaria in the WHO African Region is disproportionately high, accounting for 95% and 96%, respectively, of malaria cases and deaths in 2020. In addition, the WHO African Region documented that children under the age of five made up over 80% of all malaria deaths in 2020, making them the most vulnerable group to the disease ( Saba, Balwan & Mushtaq 2022). GBD 2019 estimated a significantly higher DALYs burden of 46.4 million years compared to WHO GHE 2019, with age-standardized rates of 667 DALYs per 100,000 (95% UI 337 – 1,150) ( Vos et al. 2020).

## Analysis of recent literature

Addressing these diseases requires up-to-date, robust, and comprehensive information on their presence, species-strain diversity, ecology, and environmental and geographical factors influencing their transmission. A targeted and region-specific approach is essential to effectively manage and mitigate their impact in Southeast Asia.

In this section, we explore the applications of ML tools in drug discovery and development, and surveillance and disease management for the aforementioned diseases. Next, we briefly discuss the advances of ML tools in adjacent fields of protein- and antibody-language models, cancer research, and computer vision that can be further leveraged for NTDs research and disease management. Lastly, we discuss steps taken for regional collaboration, data and infrastructure sharing within and around SEA and Western Pacific regions.

### Application of machine learning tools for NTDs

The conventional approach to drug discovery is expensive and takes up a considerable amount of time. Computational approaches to drug discovery using Artificial Intelligence (AI) can resolve both concerns and speed up the process of novel drug discovery. Machine learning (ML) is a subfield of Artificial Intelligence (AI) where sets of data and algorithms (mathematical and statistical) are utilized in search of distinct patterns within the data for a more efficiently accurate downstream analysis ( McComb, Bies & Ramanathan 2022). ML in drug discovery and development are carried out by looking for patterns in sets of molecules with drug- and therapeutic-properties to describe in detail their biological activities ( Dara et al. 2022). Tasks such as classification (prediction of classes), clustering (grouping of similar data items), and regression (prediction of continuous values) can be performed using ML approaches ( Oguike et al. 2022). The role of ML in limiting the spread of deadly diseases (such as disease forecasting, outbreak prediction, disease outbreak detection, and risk prediction) has been detailed reviewed by Alfred and Obit (2021).

We note that literature mining over the past five years (2017 till 2022) using Google Scholar and Public Library of Science (PLOS) on application of ‘ML tools in drug discovery and development’ resulted in articles for dengue, cysticercosis, leptospirosis, and malaria. Literature search did not return relevant articles for four other diseases, namely STHs, rabies, LF, and melioidosis. Nevertheless, literature on ‘ML tools in surveillance and disease management’ were successfully retrieved for each disease. Moreover, six of the presently listed NTDs (dracunculiasis, lymphatic filariasis, onchocerciasis, schistosomiasis, soil-transmitted helminthiases, and trachoma) can be controlled, eliminated, and prevented through recommended strategic interventions. Efforts from conducting hygiene education programs, innovative and intensified disease management, mass drug administrations (also called preventive chemotherapy), provisioning and educating the principles of safe water, sanitation and hygiene (WASH), vector control, and veterinary public health have helped speed up efforts in eliminating these diseases ( Hotez & Lo 2020; Zeynudin et al. 2022). We also suspect the possible fact that since helminthic diseases (LF and STHs in this review paper) can be easily treated with long existing anthelmintic drugs, this did not spark any interest among researchers to develop ML tools in developing a new drug against it ( Butala et al. 2021). Moreover, reports on significant decrease of LF and STHs infections were achieved through the combination of strategic interventions as recommended by WHO ( Yajima & Ichimori 2021; Zeynudin et al. 2022). In the case of rabies, there is a vaccine available to prevent the infection to both humans and animal companions along with comprehensive surveillance. Lastly, the number of melioidosis cases are comparably lower than any of the diverse group of NTDs despite the high mortality rate of the disease which could be a possible explanation on why no research employing ML tools were invested for the disease. Despite that, the DALYs burden estimates for each of the diseases underlines a pressing need among researchers to develop accurate, sensitive, and specific surveillance, clinical diagnostic, disease detecting, prediction and/or distribution modeling to reach the elimination targets for these diseases.

***Dengue***

**Drug Discovery and Development ~** The DENV replication process requires the NS3 protease domain, and the cofactor NS2b which is vital for substrate recognition and complex stability. Both molecules form the NS2b-NS3 protease complex which is a popular target candidate for antiviral drug study due to its key importance for viral replication. However, presently available choices of inhibitors during that time were unsatisfactory due to weak activity or low selective index towards the NS3 active site. The work of Aguilera-Pesantes et al. (2017) as reviewed by dos Santos Nascimento et al. (2022) utilized ML methods to identify potential residues and sites for drug-like molecule interaction, and bindable sites for drug development through the computational analysis approach for each amino acid in the DENV protease. They used four ML models, Random Forest (RF), Least absolute deviation tree (LAD Tree), voting feature interval (VTI), and multilayer perceptron algorithm (MLP), to classify their predicted data of i.) mutational susceptibility; ii.) residual binding site; iii.) physico-chemical properties; and iv.) computational alanine scanning mutagenesis for protein binding affinity and stability predictions. Each model’s performance was measured through recall, precision, area under the receiver operating characteristics curve (AUC: area under the ROC) metrics. At the end of their study, MLP-based models yielded the best performance in properly classifying residues interaction with NS3 that would cause major change in activity, moderate change in activity, and residues with similar activity as wild type residues ( Aguilera-Pesantes et al. 2017; dos Santos Nascimento et al. 2022).

The application of Artificial Neural Network (ANN) to predict Dengue-Human protein interaction type leading to development of antiviral drugs was reported by Jainul Fathima et al. (2019). They trained the model with a dataset made up of 535 non-redundant interactions between 335 different human proteins and ten dengue proteins, each of which is made up of eight attributes and 550 instances, using the Feed Forward Back Propagation Neural Network (FFBPNN) technique. As many as 12 categories of human protein interaction with dengue protein were generated to be selected for attribute selection, and then ranked based on the weights. The prediction accuracy on the test dataset was 98.05%. Two human proteins HBA1 and HSPA5 were discovered to have greater interaction with dengue virus compared to others, plus the NS3 and NS5 dengue proteins were proven to be of therapeutic drug target potential.

Khalid et al. (2020) reported a study that uses ML methods to investigate the biological activities of inhibitor derivatives anti-dengue compounds. They employed an atom-based three-dimensional (3D) QSAR modeling study along with the machine learning software Schrödinger Drug Discovery Suite Phase™ to investigate the compound's structural features with the anti-dengue activities. As a training dataset, a homologous series of 21 newly discovered 1,3,4-oxadiazole derivatives compounds was used. Using the built-in random selection mechanism of the Schrödinger Phase™ software, the prepared datasets were then separated into training and test sets, 75% and 25% respectively. Based on the predictability of the biological activities of the test molecules, the model's predictive capacity was found to be adequate with Q2 (R ^2^ Training Set) = 0.73 and Q2 (R ^2^ Test Set) = 0.78. The chemical structural features of the predicted models were used as a benchmark for developing novel 1,3,4-oxadiazole derivatives compounds against dengue virus ( Khalid, Rao Avupati & Hussain 2020). Subsequently in the same year, Geoffrey and his colleagues (2022) employed a ML-based AutoQSAR, which encompasses feature selection, QSAR modeling, validation, and prediction to generate drug leads from PubChem database for Dengue and West Nile virurs. The ML-based AutoQSAR algorithm helps to expedite virtual drug screening and identification against Dengue and West Nile virus and also perform automated *in silico* examination of the drug lead compounds. Readers interested in conducting 3D-QSAR modeling for their research are recommended to opt for Py-CoMFA, an open source web-based alternative ( Ragno 2019; Giordano et al. 2022).

Elakkiya Elumalai (2022) published a study in which ML techniques were used to identify and classify peptides as either inhibitory or non-inhibiting of the dengue virus. A dataset of 100 peptides that have been experimentally verified to inhibit the dengue virus and 16 negative datasets from the antiviral peptides database (AVPdb), were both divided into training and testing sets with a 7:3 ratio. Eight different ML algorithm models, including Adaptive Boosting (Adaboost), Bagging, k-Nearest Neighbor (kNN), Logistic Regression (LR), Multi-Layer Perceptron (MLP), Naïve Bayes (NB), Random Forest (RF), and Support Vector Machine (SVM), were used to compare three amino acid descriptors, Amino Acid Composition (AAC), Grouped Amino Acid Composition, Transition, and Distribution (GAAC), and Composition, Transition and Distribution Features (CTD). Five of their best models on training data reported accuracies greater than 85%. The same five models were used for testing in which two models (AAC_RF_model and AAC_k-NN_model) reported accuracy of 85.71%, whereas the remaining models are less than 80% accuracy. Both k-NN and RF algorithms implemented were validated as the best algorithm in achieving the research goal. In addition, their study discovered higher frequency of glycine (G), phenylalanine (F), and tryptophan (W) amino acids found in dengue virus inhibitory peptides ( Elumalai 2022).

**Surveillance and Disease Management ~** Dengue surveillance is crucial for detecting outbreaks and monitoring disease incidences. Increasing the number of surveillance traps that capture eggs (ovitraps) and ovipositing females (gravid traps) with appropriate larvicide and mosquitocide ( Selvarajoo et al. 2022). This is to prevent hatching of eggs or any subsequent production of mosquitoes inside the trap. This method is a two-pronged approach allowing authorities to survey the incidences and population of mosquitoes as well as for vector control. However, counting of both traps requires a group of individuals (insectaries) that possess considerable degree of skill to count eggs and sampling specific stages of mosquitoes ( Tsheten et al. 2021). Other than vector surveillance, insecticide resistance in the vector population should be identified via susceptibility bioassays, which should be performed by the governing authorities. Observation of the phenotypic response of mosquitoes’ post-exposure to insecticides should be a sufficient metric in determining presence of insecticide resistance.

Control of dengue is mainly achieved through the cooperation of all walks of life. Common strategies include removal of mosquitoes breeding sources, eliminating container habitats that would collect water that are favorable for oviposition sites and development of mosquito larvae, killing of larval or pupal mosquitoes by applying environmentally friendly insecticides, and usage of spatial repellents ( Srisawat et al. 2022). Various strategies of sterile insect technique (SIT) all aimed at causing decline of targeted insect population through the release of sterilized male insects were proven to be a significant measure in controlling mosquito populations ( De Castro Poncio et al. 2021; Hugo et al. 2022; Ranathunge et al. 2022). Despite these advances, dengue infection remains largely uncontrollable in both rich and poor populations due to several factors. This was seen in Singapore’s 15 year-long intensive vector surveillance from the mid-1970s to the late 1980s, where low incidences of dengue were reported. In 1990 onwards, the country faced repeating cyclical epidemics where the largest epidemic occurred back in 2013 with over 22,000 cases despite the continued investment of US$50 million in vector control annually ( Molyneux 2019). Hence, there is a need for improving active surveillance aspects and developing drugs for better disease management.

With respect to the three types of dengue infection that may manifest among patients, Hoyos, Aguilar, and Toro (2022) employed various ML decision support systems to develop an autonomous cycle of data analysis tasks (ACODAT) to help medical personnel in clinical disease management of dengue cases. Large population dataset of approximately 70,000 patients was utilized to train the tool in verifying patient data information, classifying the type of dengue a patient has contracted, and listing the best treatment accordingly. The authors used a MLP with a single layer in the case for ANN and classifier version of SVM. Both ML-based classification models achieved accurate dengue type classification (> 0.97). Then, a genetic algorithm (GA) was utilized to compute information from the classification step to generate the best treatment plan for the patient.

Two regression-based ML models of multiple linear regression (MLR) and support vector regression (SVR) were employed to predict dengue incidences using information of hospitalized dengue patients, metrological and socioeconomic ( Dey et al. 2022). From the study, the SVR models showed higher prediction accuracy of 75% with mean absolute error (MAE) value of 4.95, whereas the MLR model displayed an accuracy of 67% and MAE value of 4.57. Moreover, the models were able to show a positive correlation between the relationship of rainfall index with the incidences of dengue.

Panja et al. (2022) built an ensemble wavelet neural network with exogenous factor (XEWNet) dengue outbreak forecast model based on climatic conditions, capable of performing 75% better when forecasting short-term (26 weeks) and long-term (52 weeks) dengue incidences in subtropical regions that experience moderate to heavy rainfall throughout the year. In their study, a maximal overlapping version of discrete wavelet transformation (MODWT) algorithm in interpreting the time-dependent wavelet and scaling coefficients of rainfall and dengue interrelationship. The authors reported the statistical model employing the auto-regressive neural network (ARNN) in evaluating root mean square error (RMSE) and MAE gave the best forecast accuracies of short-term and long-term dengue outbreaks simultaneously.

Subsequently, Nguyen et al. (2022) developed a dengue fever prediction model employing the DL technique of attention-enhanced long short-term memory (LSTM-ATT) model. In Nguyen’s study, they integrated more environmental variables irrespective of time-lagged (namely evaporation, humidity, rainfall, sunshine hours, and temperature) together with DF incidences for their dengue forecast model compared to Panja’s and colleague’s work. The authors proposed LSTM-ATT to be the best performing model when compared to CNN and regular LSTM because of having integrated an additional step of attention mechanism layer right after the LSTM network step ( Zhang, Yang & Zhou 2021). When compared against the modern (CNN and LSTM) and traditional prediction models, the LSTM-ATT displayed better prediction performance in terms of low RMSE and MAE values in more than half of their geographical study locations ( Nguyen et al. 2022). When selected for outbreak prediction evaluation, the LSTM-ATT model was able to distinguish months of an outbreak from normal at an average accuracy score of 0.99, and the average sensitivity score in detecting dengue outbreak months of 0.70.

***Soil-transmitted Helminths (STHs)***

**Surveillance and Disease Management ~** Since no vectors are involved in the transmission of STHs illnesses among humans, surveillance programs can be separated into (i) prediction- and mathematical-based models covering transmission model, estimating of population at risk, predicting regions in need of MDA interventions, and (ii) active surveillance approach ( Chong et al. 2022; Mogaji et al. 2022). Important factors that determine a population's sensitivity to STHs include soil and stool samples ( Oyewole & Simon-Oke 2022). The former includes usage of algorithms and large data inputs to make precise decision making of incidences and prevalence for STHs in endemic areas. In contrast, the latter involves the expert skills of laboratory staff and technicians to conduct microscopy-based and molecular-based detection methods to detect presence of helminths and accounts for disease surveillance at the same time. Microscopy-based methods, such as the Kato-Katz and McMaster techniques, are primary diagnostic tests to detect parasites by enumerating the eggs-per-gram (EPG) metric. Although these methods are cheaper, results may vary with an increase of sample size and different survey sites ( Afolabi et al. 2022). Hence, molecular-based assays such as PCR, real-time PCR and digital PCR, loop-mediated isothermal amplification (LAMP), and cell-free DNA detection provide a more sensitive, less labor-intensive, and high-throughput detection method despite incurring additional costs for detection and surveillance programmes ( Manuel, Ramanujam & Ajjampur 2021; Vegvari et al. 2021).

Measures taken in managing cases of STHs are shared among other helminth infections. Implementing MDA programs targeting high-risk groups in endemic tropical and subtropical areas has been recognized to be effective in eliminating STH globally ( Alemu et al. 2022). In underdeveloped areas with poor facilities but endemic with STHs, adopting the principles of WASH by providing adequate sanitation, improving waste management facilities, plus public education on hygiene practices and behavioral changes targeted to populations at risk would accelerate the elimination goal ( Monnier et al. 2020). As for those diagnosed with STHs, proper provision of treatment (stronger combination of antibiotics or surgery to remove the worms) is essential in ensuring a good universal health coverage ( Abela-Ridder et al. 2020).

Identifying disease predictors for frequent incidences of NTDs co-infections (malaria and STHs) were conducted through a gut microbiota study ( Easton et al. 2020). RF models were employed to account or classify for *T. trichiura* egg counts and *P. vivax* parasitemia. A 10,000 tree RF regression model with 1,345 variables at each split was used to forecast *T. trichiura* egg counts, while a 10,000 tree RF classification model with 1,346 variables at each split was utilized to assess *P. vivax* parasitemia. Accordingly, the models reported predictor variables of transforming growth factor β and bacteria taxa when predicting for *T. trichiura* egg counts or intestinal helminth burden and incidence of *P. vivax* parasitemia, respectively. The complexities caused by the co-infection are interesting, but the authors noted that longitudinal interventional studies (antimalarial and deworming treatments) are needed to further support or validate the reported results.

Dacal et al. (2021) developed a computer vision platform that aids in quantifying *T. trichiura* infection. From 51 Kato-Katz stool sample slides containing 949 *Trichuris* spp. eggs, these images were used to train and test the CNN algorithm for automatic assessment. The algorithm showed a mean precision of 98.44% and mean recall of 80.94%. Expanding their model in identifying other helminth eggs, they included positive egg samples for both *Trichuris* spp. and *Ascaris* spp. from a co-infection individual and obtained mean precision of 94.66% and mean recall of 93.08%.

Subsequently, Ward et al. (2022) proposed an AI-based digital pathology (AI-DP) device that is tasked for automated scanning and detection of helminth eggs from fecal samples prepared via the Kato-Katz technique. Images from the Kato-Katz technique stool smear slides were collected, annotated for helminths eggs, and then continued with AI training and evaluation. The authors employed the CNN technique as well for their annotated training set. About 90% of the 16,990-image annotated STHs eggs were used to train the DL-based detection model, and then tested with the remaining 10% as test set. Overall, the AI-DP was able to achieve both weighted average precision and weighted average recall of greater than 94%.

STHs epidemiological risk modeling employing Extreme Gradient Boosting (XGBoost) and Shapley Additive explanation (SHAP) as a means of STHs surveillance ( Scavuzzo et al. 2022). A dataset of hookworm infection, environmental variables, and socioeconomic characteristics were supplied to the XGBoost model for analysis in order to model the risk of STHs infection. SHAP was utilized to understand the importance of variables for predictions in the trained model. The final XGBoost model's findings outperformed the conventional statistical models that were compared in their study based on the performance metrics of R ^2^ and Mean Square Error (MSE).

Addressing the issue of complex and time-consuming manual diagnosis of STHs infections, fuzzy c-Mean (FCM) and CNN segmentation technique (ML- and DL-based, respectively) for surveillance of human intestinal parasite ova segmentation were conducted ( Lim et al. 2022). Under the direction of parasitologists, a total of 166 pictures for each species were correctly assembled in order to train both ML-based and DL-based segmentation approaches in identifying intestinal STHs ova. Both segmentation technique models were able to accurately predict helminth species at 97% (FCM) and 100% (CNN). According to a further assessment of the segmentation identification performance (Intersection over Union, IoU) of the two models, the CNN segmentation technique yielded better results than the FCM segmentation technique approach.

***Rabies***

**Surveillance and Disease Management ~** Majority of rabies surveillance and monitoring is done by promptly identifying animals exhibiting possible rabies clinical indications, documenting the background of recently deceased companion animals, and keeping track of dog bite incidents ( Jane Ling et al. 2023). In response to the 2017 rabies outbreak that occurred in Malaysia, the authors have listed in detail rabies preventive measures, and control procedures for outbreak. Generally, the best method to control cases of rabies is to visit the nearest healthcare or veterinary services to get pre-exposure prophylaxis for both you and your companion animals. Rabies surveillance-diagnosis programs are challenging as the gold standard for rabies lyssavirus detection is direct diagnosis with brain tissue. Next, efforts in controlling the disease are troublesome when incidences of free roaming stray dogs and cats are considered a norm, plus close vicinity to wildlife habitat. Hence, most countries would only initiate a mass dog vaccination program to control the outbreak and to curb the transmission ( Molyneux 2019).

Surveillance programs for rabies were proven to be challenging as rabies-infected (rabid) animals can only be identified when clinical symptoms manifest and often lead to death. Hence, drastic actions are needed to reduce rabies mortality. As children are more vulnerable to animals, education is crucial in preventing mortalities from rabies. The death rate among children is significantly decreased by teaching people how to avoid getting bitten and what to do if they fear they have been bitten by a rabid animal. The viral load in the bite wound can be considerably reduced by cleaning it as soon as possible. To break the cycle of rabies transmission, implementing mass vaccination for dogs by health authorities has been recognized to be more cost-effective and protects the well-being of livestock and humans at the same time. As mentioned before, People at high risk of exposure to the rabies virus, such as veterinarians, laboratory workers who work closely with the rabies virus, and those who have been bitten by a potentially rabid animal, are strongly advised to get vaccinated (pre- or post-exposure prophylaxis) ( Abela-Ridder et al. 2020).

Saleh, Medang and Ibrahim (2020) conducted a comparison study on a rabies outbreak prediction model employing deep learning with long short-term memory (LSTM), a type of recurrent neural network, compared to the autoregressive integrated moving average (ARIMA) traditional algorithm model. Predictive capabilities of both models were tested against a dataset of one thousand rabies samples obtained from HealthData.com, and performance metrics were assessed based on RMSE and accuracy. The authors reported lower RMSE value (2.04) and greater accuracy (97.3%) displayed by the LSTM model, compared to the traditional ARIMA model performance of 3.12 RMSE value and only 72.1% accuracy.

In surveilling the zoonotic potential of novel viruses found in vampiric bats, Bergner et al. (2021) conducted the study while utilizing two ML models that were built by Mollentze, Babayan and Streicker (2021). Novel virus zoonotic potentials were evaluated based on a phylogenetic neighborhood model, followed by a genome composition-based model. These ML models were developed by Mollentze and colleagues employed gradient boosted machine (GBM) classifiers to predict 100 best models out of 1000 iterations ( Mollentze, Babayan & Streicker 2021). Bergner and colleagues reported the rabies virus as the only known zoonosis detected from the bats and suggested for molecular surveillance as a measure for the rabies outbreak. As published by Bergner and colleagues, they concluded the genome composition-based ML model worked best (have greater accuracy) in predicting zoonotic potential among novel viruses found in bats ( *Astroviridae, Coronaviridae, Hepeviridae, Picornaviridae,* and *Reoviridae*) as they were able to gain valuable insights of viral information allowing researchers to prioritize potentially zoonotic novel viruses compared to the phylogenetic neighborhood model.

As dogs (domestic and stray) are largely responsible for the transmission of rabies virus to humans, Thanapongtharm et al. (2021) employed a ML-based random forest algorithm surveillance study on the spatial population of dogs. The goal of the dog distribution and population RF model was to determine how dog populations and environmental and human population variables interacted. Two RF models were developed by the authors. The first (quantitative RF model) selected for grids with presence of dogs and were modeled as quantitative RF before evaluating the predictive power of the models with correlation coefficient and RMSE metrics. In the second model (binary RF model), grids were defined into presence for dogs or absence for dogs (represented by 1 and 0, respectively), and assessed the predictive power with a correlation coefficient and AUC. The models were able to accurately predict the distribution of owned dogs to stray dogs at a ratio of 6:1 (numerical figures; 12,027:1,868), with approximately 75% of the stray dogs being feral. Moreover, human population factors such as communities, human population density, and proximity to religious praying sites had a high correlation with the number of stray dogs. Association results on the population and spatial distribution of stray dogs can lighten the burden of governing bodies in better managing dog vaccination campaigns to achieve elimination targets of rabies.

***Cysticercosis***

**Drug Discovery and Development ~** A multi-epitope chimeric vaccine design study which targets *T. solium* membrane proteins reported promising results of cellular and humoral immune response stimulation, plus providing protection against both taeniasis and neurocysticercosis ( Kaur et al. 2020). Various ML algorithms were utilized in creating the vaccine. The authors used SVM modules to categorize allergic and antigenic proteins based on amino acid and dipeptide composition, the Hidden Markov model to predict B-cell epitope antigenic determinants, and ANN to detect proteasomal C terminal cleavage and T-cell epitopes. Five suitable cell membrane peptides were reported from their vaccine study, capable of stimulating required immune responses against taeniasis and neurocysticercosis.

**Surveillance and Disease Management ~** A virtual meeting convened by WHO aimed at reviewing existing diagnostic tools for *T. solium* before implementing them as public health programs to control the disease ( World Health Organization 2022a). In the context of a programmatic surveillance, the WHO recommended inclusion of specific communities or villages that are of a wider geographical area. Purposive sampling should be implemented to target high-risk humans and pigs’ interactions when the community lives in close contact with pigs or pigs roam freely where sanitation is inadequate. All means of diagnostic mapping and monitoring of *T. solium* presence in humans and pigs did not achieve the required sensitivity due to confirmatory methods via microscopy (humans), meat inspection, and serology testing. Diagnostic tests for public health programs are not well-suited as they are not commercially available and of unsatisfactory sensitivity and/or specificity. Due to the low sensitivity of currently available tests, the WHO urged for an appropriate response even if it’s a weak signal of prevalence detected. Preventive chemotherapy (PC) interventions should be initiated with confirmation of key risk factors of roaming pigs and inadequate sanitation.

According to WHO, it is possible to completely eradicate cysticercosis as a public health issue. as interventions in disease management are feasible and achievable. Beginning with *T. solium* in pigs, improving the well-being quality and management of pig husbandry can effectively break the transmission cycle. Such actions are such as vaccination programs, anthelmintic treatment for pigs, and proper set up of enclosure habitats to prevent access to human feces. To curb incidences of taeniasis and cysticercosis among humans, practice of proper WASH principles along with improved food safety and hygiene standards, as well as sufficient sanitation for the safe disposal of excrement would significantly reduce chances of contracting the diseases. In addition, community health education, MDA interventions, and appropriate case management for taeniasis (medical prescriptions) and NCC (surgery) would aid in removing the disease status as a public health concern ( Abela-Ridder et al. 2020).

A study on the cysticercosis diagnosis in pigs using proteomic information from tissues rich in antigens, which was assessed using the leave-one-out cross validation method ( Navarrete-Perea et al. 2017; Garcia et al. 2020). Protein extraction and purification experiments were conducted for the extracted *T. solium* cysts from the central nervous system and skeletal muscles of infected pigs. Generated proteomic information was then used to train the ML-based model in distinguishing between cysticercosis infected and uninfected pigs. The ML-based model was able to successfully classify cysticercosis infected pigs from non-infected ones, plus displaying similar results to the complex crude cysts extracts technique. When testing their model on human cysticercosis patients, they reported satisfactory performance of their model but noted that the model’s sensitivity was only at about 75%, and its performance could be improved with a new protein mixture dataset suited for human diagnosis.

Greater number of free-roaming pigs have a positive correlation with the transmission rate of infectious diseases, while not knowing the activity that the pigs had gone through. In tackling such matters, a body harness monitoring device has been developed and tasked in reporting the location and activity of free-roaming pigs which can provide substantial information in understanding where and how the pigs might have gotten the infection plus identifying the place of infection ( Haladjian et al. 2017). Here, three ML models (linear discriminant, KNN, and SVM) were employed to classify the activities (walking, eating, and resting) of free-roaming pigs and were then validated through the 10-fold cross validation technique. The authors noted the SVM-model outperformed the other two ML-based models with accuracy, precision, and recall of 95.8%, 75.4%, and 86.6%, respectively.

***Lymphatic filariasis (LF)***

**Surveillance and Disease Management ~** Establishment of GPELF by the WHO had set the goal in managing the transmission of LF infections via MDA of anthelmintics and alleviating the sufferings of people affected through morbidity management and disability prevention (MMDP). Surveillance programs for LF were conducted through sentinel and spot-check community surveys. Periodic transmission assessment survey (TAS) measures the impact of MDA interventions and to determine if the level of infection decreases below a target threshold ( World Health Organization 2022b).

MDA intervention to halt the transmission of infection through WHO-recommended prescriptions of albendazole, diethylcarbamazine, and ivermectin are strategies for managing LF disease. Usage of insecticide-treated bednets is recommended as a means of vector control in household environments. Application of WASH could be used to guarantee correct sanitation procedures to limit vector breeding sites and hygiene treatment of afflicted limbs for morbidity control. As LF is known to cause significant physical impairment, health authorities must ensure essential care is given to patients. Examples include skin care, exercise, and elevation to stop the progression and severity of lymphedema, treatment for adenolymphangitis flare-ups, hydrocele surgery, and encouraging community cooperation to finish the course of treatment and deal with its physical and psychological repercussions ( Abela-Ridder et al. 2020).

Analysis of epidemiological and socio-economic data to predict LF were conducted by employing ML techniques such as classification and regression tree (RT), gradient boosting machine (GBM), J48 algorithm, JRip algorithm, logistic model tree (LMT), probabilistic neural network (PNN), and NB ( Kondeti et al. 2019). In order to eliminate biases that are present in the dataset, the authors combined socioeconomic and epidemiological data before using feature selection and gain ratio feature selection to pick out pertinent features for the prediction model. The data is then partitioned (training, testing, and validation) and experimented under the 10-Fold Cross Validation framework while applying oversampling and undersampling methods to balance the dataset. The performance of the ML-based prediction models was then assessed by sensitivity, specificity, AUC, and accuracy criteria. According to the authors, the J48-based prediction model produced an AUC value of 62% and 23 additional classification rules based on six features, whereas the NB-based prediction model produced the best sensitivity and AUC (64%) results when using gain ratio feature selection and at 400% oversampling. The development of early warning systems to better apply prevention and control measures in managing LF disease within the community are a few of the benefits from both these ML-based prediction models.

Elvana and Suryanto (2022) trained a CNN-based model with the Image Processing and Data Augmentation approach to identify parasitic worms on a dataset of 210 microfilarial images. With models like VGG-16, ResNet-50, and Inception-v3 that had previously been trained with a simple 8 Convolutional layer CNN model, the authors performed transfer learning for the CNN model. The CNN was able to recognise LF worms from digital photographs with a 70% accuracy rate, even in the presence of noisy images of blood cell images during the training process.

A recent study by Dickson et al. (2022) investigated the potential of diagnostic testing scenarios surveillance after MDA campaigns against LF in detecting transmission and prevalence of the disease using a Bayesian network framework. The effectiveness of several infection markers in detecting signs of transmission was assessed by using a Bayesian network framework using antigen- and antibody-based data (Wb123 Ab and Bm14 Ab). The algorithm's ability to compare the probability of a missed positive LF result with various diagnostic testing situations and evaluate the impact of numerous participant characteristics led the authors to choose a Bayesian network analysis in this case. The network performances were evaluated in a criteria of sensitivity, specificity, True Skill Statistics, and AUC. According to the Bayesian network model, a sizable fraction of LF-positive cases went undetected by antigen- and antibody-based tests on their own. The most sensitive indication of present or previous LF infection diagnosis came from antigen-antibody combination testing (antigen plus Bm14 Ab). Hence, to increase the sensitivity of transmission surveys and prevent sudden and premature termination of MDA campaigns against LF, the combination of antigen plus Bm14 Ab were proposed for inclusion in post-MDA surveillance.

***Melioidosis***

**Surveillance and Disease Management ~** A ML-based Raman spectroscopic assay was developed to identify *B. pseudomallei* and *Burkholderia mallei* strains ( Moawad et al. 2019; Scoffone et al. 2021). To train the algorithm, 12 *B. mallei* strains, 13 *B. pseudomallei* strains, and 11 other *Burkholderia* spp. strains were prepared. Physical recording of the *Burkholderia* spp. Raman spectra were analyzed by the SVM algorithm together with information of the principal component during the Raman spectroscopic assay preprocessing step. The SVM Raman spectroscopic assay was also trained to produce three *Burkholderia* spp. classification models of (i) *pseudomallei*- *mallei*- *thailandensis* complex from *cepacian*- *glathei*- *phytofirmans* complex, (ii) identifying species of *B. mallei*, *B. pseudomallei*, and *B. thailandensis* accurately from one another, and (iii) identifying species of joined *B. cepacian* complex and *B. glathei* from *B. phytofirmans.* In each of the models, the SVM had identified the assigned bacterial complex and species accurately (>90%), except for the identification of *cepacian*- *glathei*- *phytofirmans* complex group, and *B. thailandensis* (65%). When validating the performance and sensitivities of the SVM-based Raman spectroscopic assay with unknown Burkholderia strains, sensitivities greater than 80% were obtained.

Xu et al. (2021) developed a SVM-based model tasked in detecting clinical septicemic melioidosis infection. Obtaining their data from the human peripheral blood microarray dataset, as many as 69 patients with septicemic melioidosis and a mix total of 175 non-septicemic melioidosis (healthy, type2 diabetes, recovered from melioidosis, and septicemic from other organism) were used to train the SVM-based detection model. When testing against the instance of detecting *B. pseudomallei* from a mixed group of healthy, type 2 diabetes, and recovery dataset, the SVM classifier yielded sensitivity and specificity of 0.988 and 1.000 respectively. When testing against the instance of detecting *B. pseudomallei* from other infection dataset, the SVM classifier yielded sensitivity ranging 0.857 to 1.000, and specificity of 0.889 to 1.000. A last validation of *B. pseudomallei* detection from combination of all health data plus modified infection dataset generated mean sensitivity and specificity of 0.962 and 0.979 respectively.

***Leptospirosis***

**Drug Discovery and Development ~** Abdullah et al. (2021) studied the identification of a suitable *Leptospira* spp. multiepitope-based vaccine candidate which utilized two ML programs, namely Vaxign-ML and C-ImmSim. In his study, all protein antigens have a protegenicity score greater than 90% signifying as effective antigens for vaccine developments, and simulations from C-ImmSim showed diverse immune reactions of the vaccine construct indicating promising subunits of multiepitope vaccine candidate for immunity against *Leptospira* spp. Infections.

Vaxign-ML is a reverse vaccinology (RV) tool that uses supervised ML to predict the rank score (also known as protegencity) of bacterial protective antigens (BPAgs) using a training set of viral and bacterial antigens ( Ong et al. 2020). Out of five additional ML techniques used to develop Vaxign-ML, extreme gradient boosting (XGBoost) was found to be the most effective when using nested 5-fold cross-validation (N5CV) and leave-one-pathogen-out validation (LOPOV) evaluation methods. Set as the benchmark against five other existing programs and methods, Vaxign-EGB-ML displayed satisfactory results outperforming four programs. Final validation on external data sets of clinical trials or licensed vaccines reported ranked calculation of best top 10% BPAg candidates for 20 proteins. Next, the immune simulation study server C-ImmSim uses position-specific scoring matrices and machine learning techniques to seek peptides with epitopes and other immunological interactions ( Rapin et al. 2010; Ong et al. 2020). The program combines a mesoscopic scale simulator of the immune system with a set of agent-based class computational models to predict molecular-levels of major histocompatibility complex-peptide binding interactions and neural networks for prediction of epitopes.

**Surveillance and Disease Management ~** To investigate the spatial distribution of human leptospirosis, Mohammadinia et al. (2019) have employed the ANN, geographically weighted regression (GWR), generalized linear model (GLM), and SVM approaches to model and predict the disease based on environmental parameters of temperature, precipitation, humidity, elevation, and vegetation, which has also been further reciewed and expanded upon by Guo et al. (2023). All four models were assessed based on mean square error (MSE), mean absolute error (MAE), mean relative error (MRE) and R ^2^. The authors reported that the GWR-based model displayed the best performance in the prediction of leptospirosis, followed by SVM, GLM, and ANN. It was also discovered that temperature and humidity parameters had a great influence on the distribution of leptospirosis among humans.

Predictive risk maps of leptospirosis distribution employing SVM and MLPNN algorithms were conducted by Ahangarcani et al. (2019). With further analysis and review provided by Bradley & Lockaby (2023), they evaluated the model's performance using the Kappa coefficient and AUC metrics, they looked at the association between altitude, average humidity, average temperature, days below 0°C, land cover, rainfall, slope, and leptospirosis incidents from the previous year. Incidences of leptospirosis in prior years were positively correlated with rainfall, average humidity, and average temperature, but negatively correlated with altitude, slope, land cover, and days below 0°C. Both SVM- and MLPNN-based predictive models displayed satisfactory results with Kappa coefficient and AUC greater than 83% and 0.84, respectively.

A subsequent study in identifying the relationship between the occurrence of leptospirosis with exploratory data analysis of temperature, rainfall, and relative humidity ( Rahmat et al. 2020). The authors used an ANN method using back-propagation training, optimization of hidden layers, and hidden nodes to categorize a combination of selected features into determining the presence or absence of diseases. Performance measurement of the ANN-based leptospirosis prediction is evaluated by the model’s accuracy, sensitivity, and specificity. The ANN model produced the maximum accuracy, sensitivity, and specificity of 84.0%, 86.4%, and 79.3% when measuring the robustness of the model (AUC metric) using a randomized dataset. Additionally, it was reported that using exploratory data methodologies improved the leptospirosis predictive model's accuracy from 13.3% to 31.3%. The weekly average temperature and weekly rainfall total amount at lags of 16 weeks and 12 to 20 weeks, respectively, were found to have a significant link with the incidences of leptospirosis.

***Malaria***

**Drug Discovery and Development ~** A recent study by Mswahili et al. (2021) developed and compared the performance of five ML models to predict antimalarial bioactivities against *P. falciparum.* They trained ML models of artificial neural network (ANN), SVM, RF, extreme gradient boost (XGB), and LR over a data set of 4,794 antimalarial drug candidate compounds (2,070 active and 2724 inactive molecules). The Recursive Feature Elimination (RFE) wrapper-based algorithm that treats feature selection as a search problem and the K-best filter-based algorithm that selects potential features according to a particular function were chosen as feature selection algorithms for performance examination and comparison. K-best was adopted as an accuracy metric whereas RFE was viewed as an efficiency metric. Based on the two metrics, they found that XGB, ANN, and RF models gave the best three accuracies in finding new antimalarial drug formation without losing too much precision.

Apichat Suratanee and colleagues reported the use of four ML classification algorithms, namely NB, NN, RF, and SVM, to investigate protein-protein interaction (PPI) networks for human and malarial parasites retrieved from STRING database (version 11.0), in order to identify new human proteins associated with malaria as a means of developing additional drugs against the disease ( Suratanee, Buaboocha & Plaimas 2021). A total of 12,038 human proteins with 313,359 interactions and 1,787 *P. vivax* proteins with 11,477 interactions were used to train the ML models. While examining the five topological features, including (i) betweenness centrality, (ii) closeness centrality, (iii) degree, (iv) eccentricity, and (v) Kelinberg's hub centrality, they built a heterogeneous network connecting human-human protein interactions and *P. vivax*- *P. vivax* protein interactions with the human- *P. vivax* protein associations. Next, they applied ten 10-fold cross-validations to each algorithm to produce performance metrics of a ROC curve with an AUC, with the RF method coming out on top (AUC of 0.85) and being followed by the NN, SVM, and NB algorithms (AUCs of 0.79, 0.77, and 0.74 respectively). By calculating the top-ranking score for each human protein using the RF classifier's greatest performance and results, the authors were able to acquire 411 human proteins. Subsequent functional annotation of the proteins revealed previously reports of promising candidates for multistage targets for malaria therapy.

Utilizing solely the peptide sequence data, an interpretable scoring card system was employed to pinpoint the antimalarial activity ( Charoenkwan et al. 2022). The authors trained an iAMAP with SCM-based predictor with eight other conventional supervised classifiers of decision tree (DT), k-nearest neighbor (KNN), logistic regression (LR), multilayer perceptron (MLP), naïve Bayes (NB), partial least squares regression (PLSLR), random forest (RF), and support vector machine (SVM) with nine conventional feature descriptors namely amino acid index (AAindex), PCP, amino acid composition (AAC), composition, transition and distribution (CTD), CTD-composition (CTDC), CTD-distribution (CTDD), CTD-trancomposition (CTDT), dipeptide composition (DPC), and tripeptide composition (TPC). The training data set consists of 139 positive and 2,135 negative molecules and the test set of 139 positive and 677 negative molecules. The 10-fold cross-validation approach was used to refine the program after initially estimating the propensities of 20 amino acids and 400 dipeptides. Then, estimated propensities were used to choose significant physicochemical features, and the 400 dipeptides' best propensities were used to construct the final prediction model (iAMAP-SCM). Scoring based on maximum accuracy (ACC) and Matthew’s coefficient correlation (MCC), iAMAP-SCM was reported to achieve scores of 0.957 and 0.834 respectively and outperformed the other three classifiers employed, when the model was screened independent test datasets for validation. The application of AI and ML methods to drug discovery and development of malaria have also been reviewed in several recent papers ( Oguike et al. 2022; Winkler 2021).

**Surveillance and Disease Management ~** Classification of clinical malaria using ML approaches based on hematological parameters is beneficial in improving disease management ( Morang’a et al. 2020). The authors employed six approaches to accurately categorize malaria outcomes into uncomplicated malaria, non-malarial infections, and severe malaria. ML algorithms in this study includes ANN, DT, multiple adaptive regression splines (MARS), partial least squares logistic regression (PLSLR), RF, and SVM were all fine-tuned into their best performance state according to the algorithm's kernel. ANN algorithm was further developed into three different models used for multi-classification (among all three malaria categories) and two binary classification (between two malaria categories) of the three categories, and then proceeded with RF algorithm for confirmation of clinical malaria category classification. To measure the performances of each model, metrics such as the accuracy, AUC, confusion matrix, F1 score, and precision and recall were used. All the ML-based models were able to accurately (0.78 to 0.86) classify clinical malaria from non-malarial infections, with SVM and ANN generating the best overall classification outcome. According to an assessment of ANN classification models for clinical malaria, it can distinguish between simple malaria, non-malarial illnesses, and severe malaria with an accuracy better than 0.8 and diagnostic capacities (measured by AUC) greater than 0.86. The three models created by ANN were subjected to RF analysis, which revealed that all three models had accuracy levels higher than 0.76. Platelet counts and red blood cell counts were found to be the most crucial features for categorizing the clinical malaria categories.

Okagbue et al. (2021) employed six ML algorithms, namely Adaboost, DT, kNN, LR, NN, and RF, to build malaria diagnosis models using a sample size of 337 composed of age and sex data, and 15 disease symptoms. Performance of all six models in correctly classifying positive-for-malaria from negative-for-malaria, are as follows; 68.2% (LR); 71.8% (kNN); 89.6% (DT); 92.6% (RF); 95.8% (NN); and 100% (Adaboost). Investigating the effects of including age and sex data on the performance of all six classification models, a second run solely utilizing 15 symptoms was conducted by the authors. A slight decrease in precision and accuracy performance were noted across all models. Performance for all six models remains unchanged with Adaboost being the best performing model and LR performing the least, as ranked previously in ascending order. Here, the Adaboost-based model was still the best performing model with classification accuracy of 98.2%, precision of 96.6%, and error rate of 1.8%. The authors noted that the Adaboost-based model agrees prediction accuracy from similar studies (Alzheimer’s diagnosis from MRI scan, breast cancer diagnosis, diabetes diagnosis, and prostate cancer diagnosis), and results outcome with the inclusion of sex and age data generate better AUC metrics with zero error-rate (misclassification of positive- or negative-for malaria).

A recent study employing ML- based models to predict risk for malaria based on mutation location were published by Tai and Dhaliwal (2022). In this research, researchers examined genetic variation data from 20,817 people from the Malaria Genomic Epidemiology Network (MalariaGEN). Based on 104 feature sets of malaria genetic markers, three ML-based models of LightGBM, Ridge Regression, and SVR were built to predict malaria risk. LightGBM was the top-performing ML-based model (MAE score of 6.39E-01 on all 104 features), and it also outperformed the other two ML-based models when it came to performance results when predicting with fewer features information. Additionally, 50% fewer features (52 features are enough to replace the 104 features used in the previous malaria risk prediction) were reported to be sufficient in predicting malaria risks.

In summary, nationally representative survey programs suited for the geographical and environmental etiological factors for each respective country. For example, DHS or demographic and health surveys, could provide a suitable platform for ongoing disease surveillance programs. Picking the proverb “prevention is better than cure”, core strategic interventions together with disease management will better facilitate in eliminating the prevalence and transmission of diseases, and at the same time decrease the morbidity and mortality inflicted.

### Comparisons with ML application in cancer research, computer vision, protein language models

The majority of studies employing ML models to discover novel drug candidates for NTDs (but not all), have been published in the past two decades. Similarly, applications of ML in cancer research have been in practice since the early 2000s ( Bertsimas & Wiberg 2020). Research domains where ML-based methods can be employed in cancer biology includes genomics, proteomics, metabolomics, epigenetics, transcriptomics, and system biology ( Kourou et al. 2021; You et al. 2022). In the computer science bibliography of the Digital Bibliography and Library Project (DBLP) and the biomedical repository PubMed, literature mining on ML-based studies on cancer diagnosis, patients' classification, and prognosis (excluding reviews and technical reports) between 2016 and 2020 were able to retrieve 921 and 165 studies, respectively.

Despite the advances in chemotherapy and immunotherapy, early detection of cancer increases one’s survival rate tremendously. Technological innovations have sprouted a new branch of AI known as computer vision (CV), which will significantly lighten the burden of physicians and radiologists when it comes to interpreting an MRI or histology slide for the presence of a tumor. Successful early diagnosis of breast cancer using convolutional neural network (CNN) to analyze histopathological images were reported, with additional validation from other researchers of promising and accurate diagnostic capabilities in analyzing imaging slides with the use of deep CNN architectures. Compared to detection at later stages, the cancer would have metastasized, spread to vital organs where surgery may not be feasible. As reviewed by Kourou et al. (2021), most cancer ML-based studies on cancer detection and diagnosis centered around developing DL architectures of automated diagnostic models aiding radiologists and physicians to handle and better identify or characterize imaging data (input) from computed tomography (CT), magnetic resonance imaging (MRI), X-ray radiography, and positron-emission tomography (PET). Another short example is the development of an image-based lung cancer detection model, whereby a region-based CNN model trained with 42,290 whole-CT lung scans has outperformed the average radiologists at malignancy risk-prediction and achieved AUC score greater than 95% when validated with 1,139 clinical cases ( Ardila et al. 2019; Syed & Khan, 2022). An overview of the application of AI in identifying cancer targets and drug discovery has been reviewed ( Alqahtani 2022; Shao et al. 2022; Taylor 2020; You et al. 2022).

Advances in the field of AI, typically ML and DL methods, were utilized to develop language models to predict proteins. Algorithms from these methods were employed to process the efficiency and quality of the natural language processing (NLP). To develop a protein language model (PLM), large text (protein sequences from large databases) is given as input to train the prediction of masked or missing amino acids ( Bepler & Berger 2021; Ofer, Brandes & Linial 2021; Rives et al. 2021). Literature findings on advances of protein embeddings displayed great performances in predicting secondary structure and subcellular location comparable to other methods that employ evolutionary information from MSA inputs, substituting sequence similarity for homology-based annotation transfer, and predicting mutational effects on protein-protein interactions (PPI) ( Alley et al. 2019; Heinzinger et al. 2019; Littmann et al. 2021; Stärk et al. 2021; Zhou et al. 2020).

Variant Effect Score Prediction without Alignments (VESPA) is able to predict sequence residue conservation and single amino acid variants (SAV) almost as comparatively accurately to other existing methods (DeepSequence, ESM-1v, and GEMME) without employing multiple sequence alignment (MSA) approach to learn more about the functional, structural, sequence conservation and evolutionary information that the organism or gene underwent ( Marquet et al. 2022). PLM-based structure prediction models, such as AlphaFold2 (AF2) and RoseTTAFold, successfully solved the atomic-resolution structure prediction problem by using MSAs and templates of related protein structures to provide the highest possible structural prediction performance ( Baek et al. 2021; Jumper et al. 2021). Evaluating the performance of MSA-based LMs in isolating coevolutionary signals encoding functional and structural constraints from phylogenetic correlations, Lupo, Sgarbossa, and Bitbol (2022) discovered less phylogenetic deterioration from MSA contact inference plus greater structural contacts accuracy compared to the Potts model despite the MSA Transformer being pre-trained with a minimized diversity dataset. Advancing the MSA approach, an end-to-end differentiable recurrent geometric network (RGN2) for structure prediction of single protein sequences outperforming MSA-based tools (AF2 and RoseTTAFold) was published by Chowdhury et al. (2021) which employed: (i) AminoBERT PLM, which learns latent structural information from millions of misaligned proteins; and (ii) geometric modules representing the Cα backbone geometry. Subsequently, Lin et al. (2022) developed a similar program named ESMFold capable of competing with AF2 and RoseTTAFold in atomic level protein structure prediction accuracy solely with information on individual sequences of rare proteins. Absence of MSA-based elements have significantly shortened the process of protein structure prediction as much as up to six-fold faster than existing tools.

Antibodies represent a unique group of proteins, development of an antibody language model (ALM) for prediction would definitely outperform a trained protein language model that covers a holistic range of protein. ALM AbLang outperformed both IMGT germlines and protein language model ESM-1b in terms of a faster completion time and capability in restoring missing residues of antibody sequences retrieved from the Observed Antibody Space (OAS) database ( Olsen, Moal & Deane 2022). Ablang was able to scrutinize better by separating the sequences based on V-genes into smaller clusters, segregate and classify accurately between naïve and memory B-cells, and restored missing residues of sequences with 15 missing residues at the N-terminal without any additional germlines information. Another antibody LM, Antibody-specific Bidirectional Encoder Representation from Transformers (AntiBERTa), was reported to outperform two existing PLMs (ProtBert and Sapiens) and exhibited better B cell receptors representation when compared to ProtBERT that was assigned with a smaller dataset ( Choi 2022; Leem et al. 2022). The authors described self-attention changes as the element of AntiBERTa allowed it to correctly predict paratope positions of both CDR and non-CDR positions, better distinguish naïve and memory B-cells than two other PLMs and focused on what is functionally important for specific binding. Interested readers are referred to the design methods of a linguistic-based formalization of the antibody language ( Vu et al. 2022). Readers interested in methods of antibody language models are referred to a comprehensive review that discuss the progress, methods, and challenges ( Akbar et al. 2021, 2022).

In summary, the applications of ML tools in both neglected diseases and cancer, computer vision, and protein language research are very similar with one another. Both fields utilized ML tools for drug discovery and development, novel drug target sites, disease surveillance, prediction, detection, and disease management. The main factor that gaps both fields from one another is that researchers are more invested in advancing breakthroughs in cancer, computer vision, and protein language. Moreover, this research have been primarily driven by the accumulation of large training datasets and the development of highly sophisticated deep learning architectures. Typically, large attention-based models are trained on datasets in the order of 10 ^6^–10 ^7^ data points. In contrast to NTD research where datasets remain restricted at the scale of 10 ^2^–10 ^3^ (up to five orders of magnitude lower), shallow ML-methods, namely LR, RF, SVM, among others, are more prevalent. The lack of large datasets restricts the widespread application of large deep learning models for the discovery of new NTD therapeutics and thus hampers the potential for efficient management and eradication of these diseases.

### On regional collaboration, data, and infrastructure sharing

The Southeast Asian countries are strategically located and exceptionally diverse in culture. Apart from the geographic proximity of Member States of the ASEAN regional association, the countries share a few other similarities of having densely populated communities, mineral-rich economies that open throughout the globe, and share similar tropical and subtropical climates. Hence, when there’s a disease outbreak reported among any of the SEA countries, chances of the imported disease to neighboring countries are very high. Hence, there is a need to circumvent the matter through regional collaboration, and data plus infrastructure sharing among the SEA countries.

As previously described, the SEA region is endemic to vector-borne diseases such as arboviral diseases (dengue, LF, and malaria), leptospirosis, cysticercosis, and rabies. These diseases require up-to-date, robust, and comprehensive information on presence, species-strain diversity, ecology, environmental and geographical information regarding the organisms that carry and transmit the infectious agents. As such, a few platforms had been established, all aimed at tackling these vector-borne diseases at a regional scale, may it be as an open-access publicly available database, or an online platform aimed at collecting data, analyzing them, and providing technical skills and collaboration with both local and global stakeholders. Two very notable and frequently visited database for health metrics and disease related data retrievals that have been actively mentioned throughout this review are none other than WHO’s Global Health Observatory (GHO) and Global Health Data Exchange (GHDx) data catalog that is created and supported by the Institute for Health Metrics and Evaluation’s (IHME) at the University of Washington, where both the Global Health Estimate (GHE) 2019 data and Global Burden of Disease Study (GBD) 2019 could be retrieved respectively by interested readers. Next, the European Centre for Disease Prevention and Control (ECDC) is an open-access database on dengue surveillance, threats, and outbreaks governed by the European Union. The database consists of almost all NTDs and other diseases that are of public health concern. Like MAP, ECDC serves as a platform that collects, analyses, shares, and provides infectious disease data and guidance as a means of assessing disease risks, preventing, and responding to outbreaks and other public health threats. Another publicly available but somewhat geographically irrelevant to SEA region is the WHO-driven Expanded Special Project for the Elimination of NTDs (ESPEN). However, the ESPEN portal only contains survey data sets of NTDs in Africa in response to 39% of the global NTDs burden that occurs in Africa. Undeviating from the WHO goal of accelerating the elimination and eradication of NTDs, ESPEN serves as a portal to aid governing bodies and health officials to rapidly strategies and deploy NTDs interventions to reach key targeted communities.

The Global Alliance for Rabies Control (GARC) is the leading international non-profit organization dedicated to eradicating canine rabies. GARC collaborates with global stakeholders, governments, and local partners to increase public awareness of rabies, promote teamwork, and develop the data required to increase political commitment and funding. Their main team of nine members work across three established work networks of ARACON (Asian Rabies Control Network), MERACON (Middle East, Eastern Europe, Central Asia and North Africa Rabies Control Network), and PARACON (Pan-African Rabies Control Network) as an effort to end rabies. International body responsible for infrastructure sharing in combating LF is the Global Alliance to Eliminate Lymphatic Filariasis (GAELF). Having great breakthroughs by GPELF in eradicating LF as mentioned previously, GAELF is a steering body aimed at bringing relevant partners to support the GPELF established by WHO via political, financial and technical resources mobilization. The Global Atlas of Helminth Infections (GAHI) is an open-access database that details the global and geographical distribution of three neglected tropical worm-borne diseases: LF, STHs, and schistosomiasis. The platform was developed by London Applied & Spatial Epidemiology Research Group (LASER) at the University of London. All GAHI resources are available on an open access basis but up till the year 2015 only.

In response to the neglect of melioidosis, there is an active website Melioidosis.info which serves as an online-platform for reporting melioidosis cases and for disseminating information of melioidosis for the public, researchers and health policy makers. Researchers or health authorities with culture-confirmed melioidosis cases and deaths, backed with institutional support can contribute to the platform by uploading melioidosis cases and serological information. The team’s aim is to allow local and global policy makers to easily pinpoint incidences of melioidosis cases geographically and to identify institutions that are capable of diagnosing the disease. In response to the outbreak of leptospirosis, Global Leptospirosis Environment Action Network (GLEAN) was established to lessen the impact of leptospirosis on the planet by improving our knowledge of the connections between the disease's occurrence and various associated factors, such as biological, demographic, environmental, ecological, and economic factors, delivering more prompt warnings of outbreaks, and identifying prevention and control strategies. In order to predict geographical limitations, prevalence, and endemicity of malaria in every region of the world, the Malaria Atlas Project (MAP) is an open-access database and the WHO collaborative platform for geospatial disease modeling. Solely by handling data, collaboration, analytics, and engagement elements by members of MAP, they have successfully helped (i) to generate malaria mapping risk (infection prevalence, incidence rates, and mortality estimations) at national and global levels; (ii) forecast annual global malaria burden; (iii) tracked interventions coverage (malaria drugs, diagnostics, and vector control); (iv) employed statistical models to measure the effectiveness of currently available control strategies against malaria; (v) developing tools to support efficient commodities planning to ensure enough resources were prepared to protect a population; and (iv) strengthening skills among researchers and technicians in malaria analytics.

Data and infrastructure sharing is undeniably crucial for NTDs since the scale of publicly available datasets for NTDs research is restricted. Efforts displayed by each governing body in maintaining and keeping up-to-date open-access databases or infrastructure and technical outreach organizations are to ensure that every country would have the latest disease intelligence and technical skills in order for effective surveillance, preventive, and disease management control to be executed according to each country’s governing leadership. Importance of having centralized data sharing at a regional scale has been highlighted in a study by Alemu et al. (2022). With access to publicly available standardized survey and treatment coverage data, which was at first unavailable probably due to absence of reports by the country’s Ministry of Health to the WHO, they were now able to access ample amounts of collected evidence pointing to the advantages of school-based deworming programs and LF MDA campaigns.

## Conclusion

NTDs impact nearly 2 billion people especially in countries with developing economies such as countries in Southeast Asia region causing reduction in productivity and substantial accumulation of Disability-Adjusted Life Years, DALY. Machine learning has been widely applied in the fields of NTDs for drug discovery and development, plus surveillance and disease management. However, the application of machine learning in NTDs research is hampered by the limited amount of data, the absence of centralized/standardized collaborative framework and the general lack of attention from public and private stakeholders alike when compared to other fields. Melioidosis, leptospirosis and malaria, although excluded from the NTD list, continues to pose significant challenges in the region, warranting further consideration for inclusion in the NTD category. To unleash the full potential of machine learning to elevate the state-of-the-art NTDs surveillance, management, and treatment, increased investment in terms of research funding, public-private collaborative initiative, data accumulation and sharing are desperately needed. Addressing the three other diseases of public health concern alongside other NTDs in this manner would enhance disease management and mitigate its longstanding burden in vulnerable communities.

## Data Availability

No data is associated with this article.
