# Supplementary material for: Progress and challenges for the application of machine learning for neglected tropical diseases
Source: F1000Res. 2025 Jul 17;12:287. Originally published 2023 Mar 15. [Version 3] doi: 10.12688/f1000research.129064.3 (PMC12242132; doi:10.12688/f1000research.129064.3)
Supplement: Peer review history

# Reviewer report: Reviewer response for version 3

**DOI:** 10.5256/f1000research.184903.r398896

**Published:** 2025-07-23

**Authors:** Erma Sulistyaningsih

**Affiliations:** 1 Department of Parasitology, University of Jember, Jawa Timur, Indonesia

Dear authors,

 Thank you for your clarification and added comprehensive discussion on NTDs.

Is the review written in accessible language?

Yes

Are all factual statements correct and adequately supported by citations?

Yes

Are the conclusions drawn appropriate in the context of the current research literature?

Yes

Is the topic of the review discussed comprehensively in the context of the current literature?

Partly

Reviewer Expertise:

molecular tropical medicine especially in parasitology.

I confirm that I have read this submission and believe that I have an appropriate level of expertise to confirm that it is of an acceptable scientific standard.

# Reviewer report: Reviewer response for version 3

**DOI:** 10.5256/f1000research.184903.r398897

**Published:** 2025-07-23

**Authors:** Karla P. Godinez-Macias

**Affiliations:** 1 University of California San Diego, San Diego, California, USA

The authors addressed this author's concerns. No additional changes are needed.

Is the review written in accessible language?

Yes

Are all factual statements correct and adequately supported by citations?

Partly

Are the conclusions drawn appropriate in the context of the current research literature?

Yes

Is the topic of the review discussed comprehensively in the context of the current literature?

Yes

Reviewer Expertise:

bioinformatics, neglected diseases, drug discovery

I confirm that I have read this submission and believe that I have an appropriate level of expertise to confirm that it is of an acceptable scientific standard.

# Reviewer report: Reviewer response for version 2

**DOI:** 10.5256/f1000research.179710.r394708

**Published:** 2025-07-09

**Authors:** Karla P. Godinez-Macias

**Affiliations:** 1 University of California San Diego, San Diego, California, USA

In this review, Khew et al. describe the use of machine learning in neglected tropical disease (NTD) and how it can be utilized to improve surveillance and treatment of neglected tropical diseases. This review provides a cohesive overview of neglected diseases defined by WHO, machine learning advances in various fields and how it has been applied to neglected diseases surveillance. Authors also discuss the current implications of machine learning, and thoroughly describe the various AI models available and what their outcomes are in terms of disease understanding and advances.

 In this reviewer opinion, the suggested minor changes are:

1. It would be helpful to provide few examples to support some statements throughout the manuscript. For example, it is not clear which diseases are contributing to “149 countries and with more than 1.7 billion of infected individuals”
2. The line “thus greatly contributing to the spread of NTDs among women and children” (under DALY impact section) suggest that NTDs only affect women and children. Please rephrase to improve clarity and provide the corresponding citation.
3. Please consider replacing some casual phrasing (e.g., runner-ups, picked up) to a formal writing. This will drastically improve the tone and clarity throughout the text.
4. There are several minor grammatical errors throughout the text, please revise and correct them. Also, please check for consistency with the use of parenthesis and other punctuation markers (e.g., roman bullet points).
5. The second paragraph under “application of machine learning tools for NTDs” describes the literature of the past five years but describe results from 2017-2022 (8 years ago); I suggest revisiting this paragraph because literature has changed since that period. It will also be helpful to confirm the searches still hold; the use of machine learning has become very popular and some claims from this paragraph needs to be adjusted.
6. Could you please mention which NTDs do not have published studies with ML predicted drug targets (first paragraph under comparison with ML application in cancer research, …). Also, could you please update the number of studies in the repositories to reflect the past 5 years (search between 2020 – 2025).
7. Please add the missing citations to databases/datasets mentioned throughout the manuscript, if available. This is important to acknowledge the contributions of the original authors.
8. The conclusion describes the current implications of ML. It would be helpful to add a paragraph to summarize the main points of this review (e.g., current diseases status, ongoing work to tackle them), as well as some of the current and potential limitations of using ML in neglected diseases.

Is the review written in accessible language?

Yes

Are all factual statements correct and adequately supported by citations?

Partly

Are the conclusions drawn appropriate in the context of the current research literature?

Yes

Is the topic of the review discussed comprehensively in the context of the current literature?

Yes

Reviewer Expertise:

bioinformatics, neglected diseases, drug discovery

I confirm that I have read this submission and believe that I have an appropriate level of expertise to confirm that it is of an acceptable scientific standard, however I have significant reservations, as outlined above.

# Reviewer report: Reviewer response for version 1

**DOI:** 10.5256/f1000research.141719.r215469

**Published:** 2024-05-15

**Authors:** Erma Sulistyaningsih

**Affiliations:** 1 Department of Parasitology, University of Jember, Jawa Timur, Indonesia

I found some references are more than 5-years old publications (2015, 2012, 2013, 2016).

 The manuscript is about the application of machine learning for Neglected Tropical Diseases (NTDs), the authors already mentioned the list of 20 diseases of NTDs recognized by WHO (Table 1), however, the authors also write on malaria in the next session. Please specify the discussion on the 20 NTDs.

Is the review written in accessible language?

Yes

Are all factual statements correct and adequately supported by citations?

Yes

Are the conclusions drawn appropriate in the context of the current research literature?

Yes

Is the topic of the review discussed comprehensively in the context of the current literature?

Partly

Reviewer Expertise:

Molecular parasitology, molecular medicine

I confirm that I have read this submission and believe that I have an appropriate level of expertise to confirm that it is of an acceptable scientific standard, however I have significant reservations, as outlined above.

# Response

**Published:** 2024-05-24

**Authors:** Ethan Khew

**Affiliations:** The National University of Malaysia, Malaysia

Dear Erma Sulistyaningsih,

 Thank you very much for your valuable feedback on our manuscript. We appreciate the time and effort you have taken to review our work and provide constructive comments.

 Regarding the use of references older than five years, we selected these sources intentionally as many of them are seminal works or original studies that first addressed key issues (e.g., global distribution burden studies). These foundational papers are frequently cited by more recent publications, and we believe it is more ethical and academically honest to acknowledge the original sources. However, we understand the importance of including up-to-date references, and we will review our citations to ensure a balanced representation of both seminal and recent works. We will also add perspectives from recent publications, but we have noted that there is a lack of new research or evaluation on certain topics (such as disease burden), leading us to rely on older, yet still relevant, publications.

 Concerning the inclusion of malaria in our discussion, we recognize that this might appear to deviate from the list of 20 NTDs recognized by the WHO. Our review is specifically focused on the Southeast Asia and Western Pacific regions, where there is a limited number of machine learning-based studies on the recognized NTDs. To provide a comprehensive overview, we expanded our scope to include other significant diseases such as malaria and melioidosis, which are of considerable concern in these regions. Both diseases, we believe, merit attention and potential inclusion in the NTD list due to their impact. We apologize for any confusion this may have caused and are open to amending the manuscript to clarify our rationale and align more closely with the recognized NTDs if that would address the concern.

 We hope this clarifies our decisions and are ready to make necessary adjustments to improve the manuscript once we hear back from you. Thank you once again for your insightful feedback.

 Best regards,

 Ethan Khew

# Reviewer report: Reviewer response for version 1

**DOI:** 10.5256/f1000research.141719.r208370

**Published:** 2023-10-10

**Authors:** Anupam Nath Jha

**Affiliations:** 1 Department of Molecular Biology and Biotechnology, Tezpur University, Tezpur, Assam, India

The authors have written about different NTDs and machine learning techniques in the review article.

 Different types of research works are done and reviewed on neglected tropical diseases (e.g. Jha, 2023 ^1^). Here the authors have listed all such diseases and explain the causing agent. Further the application of advance machine learning approaches in drug discovery and development of such diseases are elaborated.

 It is good to read articles about the present status of NTDs but the authors are suggested to add the future scope of different computational approaches in surveillance, management and treatment of these diseases. Also, the limitations of people working in this field should be mentioned because limited data is not the only problem in handling NTDs.

Is the review written in accessible language?

Yes

Are all factual statements correct and adequately supported by citations?

Partly

Are the conclusions drawn appropriate in the context of the current research literature?

Partly

Is the topic of the review discussed comprehensively in the context of the current literature?

Yes

Reviewer Expertise:

Computational Biology

I confirm that I have read this submission and believe that I have an appropriate level of expertise to confirm that it is of an acceptable scientific standard.

## References

[1] : Editorial: Computational approaches to build therapeutic paradigms targeting genes, proteins and pathways against neglected tropical diseases (NTDs). *Frontiers in Genetics* .2023;14: 10.3389/fgene.2023.1183034 10.3389/fgene.2023.1183034 PMC1023030237265962
